# Trypsinogen Pro‐Region as a Molecular Scaffold for the Design of Competitive Peptide Inhibitors of Trypsin‐Like Proteases

**DOI:** 10.1002/arch.70224

**Published:** 2026-10-04

**Authors:** Geisiane Aparecida Mariano, João Vitor Aguilar de Oliveira, Rafael Júnior de Andrade, Daniel Guimarães Silva Paulo, Yaremis Beatriz Meriño Cabrera, Eulálio Gutemberg Dias dos Santos, Ian Lucas Batista Santos, Milena Godoi Lima, Maria Julia Summy, Otávio José Bernardes Brustolini, Neilier Rodrigues da Silva Júnior, Humberto Josué Oliveira Ramos, Maria Goreti de Almeida Oliveira

**Affiliations:** ^1^ Laboratory of Enzymology and Biochemistry of Proteins and Peptides, Department of Biochemistry and Molecular Biology Universidade Federal de Viçosa, UFV, BIOAGRO/INCT‐IPP Viçosa Minas Gerais Brazil; ^2^ Department of Biochemistry and Molecular Biology Federal University of Viçosa Minas Gerais Brazil; ^3^ Institute of Biotechnology Applied to Agriculture—BIOAGRO‐UFV Viçosa Brazil; ^4^ Department of Entomology Federal University of Viçosa Minas Gerais Brazil; ^5^ National Laboratory for Scientific Computing Bioinformatics Laboratory—LNCC, Petrópolis Rio de Janeiro Brazil

**Keywords:** biopesticides, enzyme kinetics, insect trypsins, protease inhibitor peptides

## Abstract

Soybean (*Glycine max* L. Merrill) productivity is severely impacted by the velvetbean caterpillar *Anticarsia gemmatalis* Hübner, 1818 (Lepidoptera: Erebidae), a major defoliating pest in soybean‐producing regions. Current management strategies rely heavily on chemical insecticides, which are associated with environmental risks and the development of resistance. Protease inhibitors have emerged as a promising alternative, as protein digestion in lepidopteran larvae is primarily mediated by trypsin‐like serine proteases. In this context, the rational design of short inhibitory peptides represents a potential strategy for developing selective and environmentally safer pest control agents. This study introduces the regulatory pro‐region of trypsinogen as a previously underexplored scaffold for the rational design of competitive peptide inhibitors targeting digestive trypsin‐like enzymes in agricultural insect pests. Fifteen peptides derived from the trypsinogen pro‐region were initially *screened in silico* against *A. gemmatalis* trypsin‐like enzymes. Molecular docking analyses revealed favorable binding energies and interactions with residues located in the catalytic region. Among the candidates, the peptide Pep12 (designated GORE3) was selected for further optimization by substituting amino acids at the P1 position, yielding the analogs GORE4 and GORE5. Enzyme inhibition assays with bovine trypsin and partially purified *A. gemmatalis* trypsin‐like enzymes demonstrated that all peptides are competitive inhibitors. The inhibition constants (Ki) obtained for *A. gemmatalis* enzymes were 1.80 mM for GORE3, 1.63 mM for GORE4, and 0.78 mM for GORE5, with GORE5 exhibiting the lowest Ki among the evaluated peptides, although its inhibitory affinity remained within the millimolar range. Collectively, these findings underscore the potential of trypsinogen‐derived peptides as promising scaffolds for developing peptide‐based inhibitors targeting insect digestive proteases.

## Summary

- Trypsinogen‐derived peptides were designed as insect trypsin inhibitors.
- GORE3–GORE5 interacted with the catalytic region of digestive trypsins.
- Kinetic assays confirmed competitive inhibition of trypsin‐like enzymes.
- GORE5 exhibited the lowest K_i_ among the evaluated synthetic peptides.
- Trypsinogen pro‐regions represent promising scaffolds for peptide inhibitors.

## Graphical abstract

Rational design of the trypsinogen pro‐region generated the peptide inhibitors GORE3, GORE4, and GORE5. Computational screening and enzyme kinetics demonstrated competitive inhibition of trypsin‐like proteases, with GORE5 showing the highest experimental potency and supporting pro‐region‐derived peptides as promising protease‐targeting molecules.

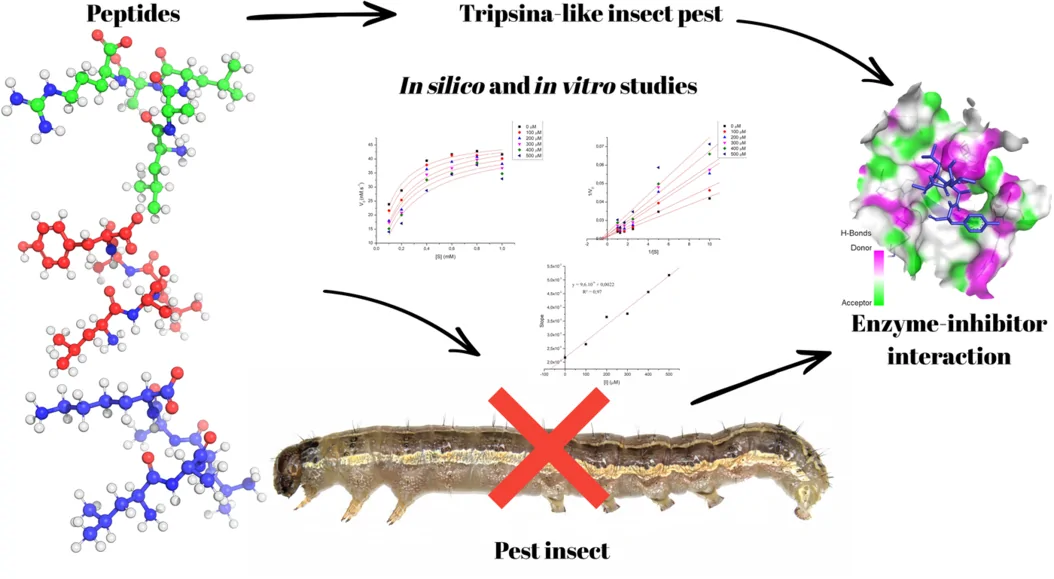

## 1 Introduction

Soybean (*Glycine max* L. Merrill) is the most extensively cultivated legume globally and is a fundamental component of worldwide food security. Continuous population growth, especially in developing regions, has heightened demand for high‐quality protein sources, underscoring the strategic significance of soybeans for their high protein content, balanced amino acid profile, and rich array of bioactive compounds. In addition to direct human consumption, soybeans are a key ingredient in animal feed formulations and serve as a major source of vegetable oil and feedstock for biodiesel production. Collectively, these qualities emphasize their significance in agronomic, nutritional, and industrial contexts within modern agroecosystems (Singh et al. 2008; Wang and Komatsu 2017; Peruca et al. 2018; Hayashida et al. 2023).

In Brazil, soybeans represent a crucial element of agri‐food systems and global nutritional security, attributable to their substantial protein and oil content, which support human nutrition, livestock feed production, and various industrial uses (Wang et al. 2023; Montanha et al. 2024; Peng et al. 2026). However, the high productivity of this crop is constantly threatened by arthropod pests, particularly the lepidopteran species *Spodoptera frugiperda* J.E. Smith (Lepidoptera: Noctuidae) and *Anticarsia gemmatalis* Hübner, 1818 (Lepidoptera: Erebidae), which are responsible for severe defoliation and significant losses in both grain yield and quality (Horikoshi et al. 2021).

The management of these pests primarily depends on the extensive application of chemical insecticides. Despite their short‐term efficacy, such compounds exert strong selective pressure on target populations, accelerating the evolution of resistance, and cause substantial environmental contamination and adverse effects on soil and water systems, non‐target organisms, and human health (Ongaratto et al. 2021; Lanzaro et al. 2024). This scenario underscores the critical importance of implementing sustainable, targeted, and environmentally compatible pest management strategies. Protease inhibitors (PIs) have emerged as promising candidates for disrupting digestive physiology by inhibiting essential proteolytic enzymes in the insect midgut. Among these targets, trypsin‐like serine proteases have received particular attention due to their central role in larval digestion.

In lepidopteran species, trypsin‐like serine proteases constitute the predominant class of digestive enzymes and are critically involved in protein hydrolysis and nutrient uptake (da Silva Júnior et al. 2020). Inhibition of these enzymes disrupts digestive homeostasis, resulting in impaired nutrient assimilation, reduced larval growth and development, and decreased survival rates. Although plants synthesize natural PIs, such as the soybean Kunitz trypsin inhibitor (SKTI), long‐term coevolution between plants and herbivorous insects has promoted the evolution of compensatory and adaptive responses that attenuate their inhibitory efficacy (Paulo, Schultz, et al. 2026). Furthermore, high‐molecular‐weight inhibitory proteins are often associated with increased production costs, structural complexity, and operational constraints that limit their feasibility for large‐scale agricultural deployment (Meriño‐Cabrera et al. 2020; de Almeida Barros et al. 2021). These limitations have stimulated the search for smaller and more selective inhibitory molecules.

Within this framework, the rational design of low‐molecular‐weight mimetic peptides represents a strategic and innovative approach. Such synthetic peptides can be tailored to selectively target the active site of trypsin‐like enzymes, potentially conferring improved stability, cost‐effective synthesis, and reduced susceptibility to compensatory adaptive mechanisms in herbivorous insects (Meriño‐Cabrera et al. 2020; Meriño‐Cabrera et al. 2019). Previous studies have demonstrated that peptides derived from functional regions of natural inhibitors or from the active site of proteases themselves can efficiently modulate the activity of digestive trypsins in lepidopteran species (Paulo, Schultz, et al. 2026; de Almeida Barros et al. 2022; de Oliveira et al. 2020; Schultz et al. 2024; Paulo, Schneider, et al. 2026).

However, despite these promising results, previously described peptide inhibitors still present important limitations, including reduced structural stability, susceptibility to proteolytic degradation, variable inhibitory potency, and limited selectivity under physiological conditions (Muttenthaler et al. 2021; Meriño‐Cabrera et al. 2022; Beatriz Meriño‐Cabrera and Goreti de Almeida Oliveira 2022). In addition, most currently available inhibitory peptides have been designed using canonical inhibitor motifs or active‐site mimetics, whereas the regulatory pro‐region of trypsinogen remains underexplored as a biologically informed template for inhibitor development (Meriño‐Cabrera et al. 2020; de Almeida Barros et al. 2021; Meriño‐Cabrera et al. 2022; Meriño‐Cabrera et al. 2020). Because this endogenous region naturally participates in protease regulation and activation control, its structural features may provide an alternative scaffold for the rational design of competitive inhibitors with distinct interaction profiles and improved target specificity.

Despite these advances, the practical application of short peptide inhibitors in insect control still presents important limitations. Due to their reduced molecular size, these peptides may exhibit lower structural stability and greater susceptibility to proteolytic degradation in the insect midgut, potentially reducing their persistence and inhibitory efficacy in vivo (Paulo, Schultz, et al. 2026; de Almeida Barros et al. 2021; Schultz et al. 2024, 2026; Paulo, Meriño‐Cabrera, et al. 2026). In addition, some mimetic peptides display lower inhibitory potency and limited specificity when compared with larger canonical protein inhibitors (Paulo, Schneider, et al. 2026). Although short peptides are generally more accessible to chemical synthesis, challenges related to formulation stability, large‐scale production, and field application remain significant barriers to their practical agricultural use (de Andrade et al. 2026; Patarroyo‐Vargas et al. 2018; Hemmati, Takalloo, et al. 2021). Consequently, computational strategies have become increasingly important for accelerating the identification and optimization of novel inhibitory candidates (Paulo, Schneider, et al. 2026; de Andrade et al. 2026).

Structural bioinformatics tools, including molecular modeling and docking approaches, have expanded the capacity to identify and optimize inhibitory candidates before experimental validation, thereby reducing the time and costs associated with conventional screening (Paulo, Schultz, et al. 2026; de Almeida Barros et al. 2021; Schultz et al. 2026; Silva‐Júnior et al. 2021; Rios‐Díez et al. 2022; Coura et al. 2022) strategies. Trypsin (EC 3.4.21.4), extensively characterized in its bovine form, represents a robust structural model for inhibitor design, particularly considering that its inactive precursor, trypsinogen, contains an N‐terminal fragment with intrinsic regulatory function. The rational exploration of these structural regions provides a mechanistic foundation for the development of peptides specifically targeted to the catalytic site. Nevertheless, the use of trypsinogen‐derived regulatory regions as templates for insect PIs remains underexplored.

The trypsinogen pro‐region naturally modulates protease activation, suggesting that its structural features may serve as a biologically informed scaffold for the development of competitive inhibitors (Beatriz Meriño‐Cabrera and Goreti de Almeida Oliveira 2022; Perona and Craik 1995). Therefore, using this endogenous regulatory segment represents an innovative approach to the systematic design of peptides that selectively inhibit insect digestive proteases (Paulo, Schultz, et al. 2026; de Almeida Barros et al. 2021; Paulo, Schneider, et al. 2026; de Andrade et al. 2026).

Considering the predominance of trypsin‐like enzymes in the midgut of *A. gemmatalis* and the pressing need for sustainable solutions for velvetbean caterpillar management, the rational design of peptide inhibitors targeting these enzymes represents a promising biotechnological strategy. Despite previous advances in peptide‐based inhibitors, the regulatory pro‐region of trypsinogen remains underexplored as a structural scaffold for inhibiting insect digestive proteases. Therefore, the present study aimed to design, prospectively screen, and experimentally validate trypsinogen‐derived mimetic peptides from *A. gemmatalis* as competitive inhibitors of trypsin‐like enzymes using an integrated approach combining structural bioinformatics and in vitro kinetic assays.

## 2 Materials and Methods

### 2.1 In Silico Identification and Design of Candidate Peptides

#### 2.1.1 Peptide Design

The pro‐region of trypsin displays intrinsic inhibitory activity, playing a key role in the physiological control of proteolytic activity (Baker et al. 1992; Jitonnom et al. 2012). Based on this characteristic, peptides comprising five to ten amino acid residues were derived from the trypsinogen sequence of *A. gemmatalis*. The amino acid sequences of non‐crystallized trypsin‐like enzymes, including the transcript‐derived sequence from *A. gemmatalis* (transcript ID: DN773_i3) and the sequences from *S. frugiperda* (NCBI accessions: XP_050552352.1, ACR25157.1, QLC28936.1, and XP_050550273.1), were retrieved from the National Center for Biotechnology Information (NCBI) database. To evaluate the conservation of the peptide‐binding site among the analyzed enzymes, sequences and structural data from previously crystallized proteins, including those of *Bos taurus* and *Homo sapiens*, were retrieved from the Protein Data Bank (PDB) (Berman 2000). The sequences were subjected to multiple sequence alignment using the Clustal Omega program (Sievers and Higgins 2014). The candidate peptide sequences were subsequently subjected to structural modeling using the PEP‐FOLD3 server (Lamiable et al. 2016). Fragments exhibiting less than 80% sequence identity and/or similarity within the aligned peptide region were deemed non‐viable and consequently excluded from subsequent analyses. This threshold was established to maintain structural and physicochemical properties that may be linked to enzyme recognition and inhibitory efficacy, while preventing undue sequence divergence among candidate peptides.

#### 2.1.2 Structural Modeling and Validation of Enzymes

For enzymes without available crystallographic structures, three‐dimensional models were generated through homology modeling using the Phyre2 server (Kelley et al. 2015; Basyuni et al. 2018). The template structures selected for each sequence were documented and incorporated into the structural validation workflow of the generated models. Model quality was assessed using multiple structural validation tools. Overall model quality was evaluated using the ERRAT program, and scores above 50 were considered indicative of structurally reliable models (Colovos and Yeates 1993). The VERIFY‐3D program was used to evaluate the compatibility between the predicted three‐dimensional structure and its primary amino acid sequence. Models were classified as high quality when at least 80% of the residues exhibited scores greater than 0.2 (Eisenberg et al. 1997). Stereochemical quality was assessed using PROCHECK, which evaluates the distribution of φ (phi) and ψ (psi) backbone dihedral angles through Ramachandran plot analysis. Models were deemed satisfactory when more than 90% of the residues were positioned within allowed regions (Laskowski et al. 1993). Additionally, structural validation was complemented by the WHATCHECK program to detect general structural inconsistencies, including symmetry‐related parameters, molecular geometry, and bond‐length deviations (Hooft et al. 1996). In this analysis, models exhibiting adequately packed residues and scores above −5 were considered structurally reliable. When a model exhibited scores comparable to or higher than those of the corresponding template structure in a specific validation test, it was deemed acceptable, even if the obtained values were marginally below the conventional cutoff criteria defined by the validation tools.

#### 2.1.3 Molecular Docking

Protein–peptide interactions between trypsin‐like enzymes and the selected inhibitors were assessed by molecular docking using AutoDock Vina implemented within the PyRx environment (Eisenberg et al. 1997; Laskowski et al. 1993). The docking grid was centered on the enzymes' catalytic region, covering the substrate‐binding pocket and the catalytic triad residues, with dimensions of 25 × 25 × 25 Å. Docking calculations were performed with an exhaustiveness value of 8, generating nine binding poses for each peptide. Peptide structures were energy‐minimized prior to docking. Homology‐modeling templates were selected based on sequence identity, structural coverage, and conservation of catalytic residues. Docking simulations were performed under default conditions, considering the protein structures as rigid and the peptides as flexible ligands. The conformations selected for analysis were those in which the peptides exhibited direct interactions with the catalytic site or adjacent regions potentially associated with substrate recognition and catalysis. For evaluation of the designed peptides, lower predicted binding energies, together with reduced peptide length, were considered indicative of greater theoretical inhibitory potential, since shorter peptides may also offer advantages in synthesis feasibility and production cost.

Theoretical inhibition constants (*K_i_*) were estimated from the docking‐derived binding free energy values (*ΔG*) using the thermodynamic relationship $\Delta G=\mathrm{RT}\ln K_{i}$, where R is the universal gas constant (1.987 cal mol^−1^ K^−1^), and T is the absolute temperature in Kelvin. Thus, *K_i_* was calculated as $K_{i}=e^{\Delta G/\mathrm{RT}}$. These values were used exclusively as theoretical estimates for comparative ranking among peptide candidates and should not be interpreted as quantitative predictions of experimentally determined inhibition constants.

For comparison and validation of the obtained predictions, docking analyses were also performed using the previously described inhibitory peptides GORE1 and GORE2 (de Almeida Barros et al. 2021), as well as the classical protein inhibitors bovine pancreatic trypsin Inhibitor (BPTI) and SKTI. The results obtained were analyzed using PyMOL to identify residues involved in intermolecular interactions and to characterize the binding pattern between the protein and the ligand.

The peptide with the lowest estimated *K_i_* value against *A. gemmatalis* trypsin‐like enzymes was subsequently truncated, yielding four shorter peptides. AutoDock Vina was primarily employed for the initial screening and comparative ranking of peptide candidates based on predicted binding energies and theoretical K_i_ values. Among the resulting truncated peptides, the peptide designated GORE3 (Paulo, Schultz, et al. 2026) was selected for further investigation. Based on this peptide, site‐directed substitutions were introduced at the P1 position of the amino acid sequence, yielding two additional peptides, designated GORE4 and GORE5. Subsequently, ClusPro 2.0 was used as a complementary protein–peptide docking approach to further evaluate the structural stability and interaction profiles of the selected complexes. The combined use of the two tools enabled cross‐validation of the predicted interactions and provided complementary information about peptide‐binding behavior.

Additionally, molecular docking between the modeled *A. gemmatalis* trypsin‐like enzyme and the selected peptides was performed using the ClusPro 2.0 server (Kozakov et al. 2017), an online platform that enables direct receptor–ligand docking. The program generates multiple interaction models grouped into clusters and assigns weighted docking scores for relative ranking. Representative models with the most favorable ClusPro scores were selected for structural analysis. These scores were interpreted only as relative ranking metrics and not as thermodynamic binding free energies. The resulting structures were visualized and analyzed for intermolecular interactions using PyMOL (Schrödinger LLC) and Discovery Studio Visualizer (Dassault Systèmes BIOVIA), thereby identifying residues that stabilize the protein–peptide complexes.

### 2.2 In Vitro Study

#### 2.2.1 Experimental Design

Caterpillars of *A. gemmatalis* were obtained from a mass‐rearing colony maintained at the Laboratory of Enzymology and Biochemistry of Proteins and Peptides, located at the Institute of Biotechnology Applied to Agriculture (BIOAGRO), Federal University of Viçosa (UFV). Upon reaching the fifth larval instar, a total of 150 caterpillars were used for enzyme extraction, following a ratio of five midguts per 1 mL of 10^−3^ M HCl (de Almeida Barros et al. 2021). Cell lysis was performed by freezing in liquid nitrogen, followed by maceration using a mortar and pestle until complete homogenization. The resulting suspension was centrifuged at 10,000*g* for 30 min at 4°C, and the pellet was discarded. The supernatant containing the enzymatic extract was collected and stored at −80°C until further analyses. Based on the previously performed in silico analyses, the peptides GORE3, GORE4, and GORE5 were selected for the in vitro study and subsequently used in the kinetic inhibition assays described in the following sections.

#### 2.2.2 Enrichment of Midgut Trypsin‐Like Activity from *A. gemmatalis*

For the purification process, the obtained supernatant (Section 2.2‐1) was initially filtered through a 0.22 μm polyethersulfone (PES) membrane filter (GVS Filter Technology). Fractions enriched in trypsin‐like proteolytic activity were obtained by affinity chromatography using a HiTrap Benzamidine Sepharose 4 Fast Flow column (Cytiva), taking advantage of the affinity of benzamidine for the S1 specificity pocket of trypsin‐like serine proteases. The column was previously equilibrated with 50 mM Tris‐HCl buffer containing 500 mM NaCl (pH 7.4). Protein elution was performed using 50 mM glycine buffer (pH 3.0) at a constant flow rate of 1 mL min^−1^. The elution profile was monitored by measuring absorbance at 280 nm using a UV–Vis spectrophotometer (Hitachi, model U‐5100). Fractions exhibiting trypsin‐like activity were pooled and stored at −20°C. Subsequently, samples were concentrated using a cellulose membrane filter with a 3‐kDa molecular weight cutoff (MWCO; Merck). Fractions obtained after purification and concentration were analyzed by one‐dimensional SDS‐PAGE (Kozakov et al. 2017), using a 12.5% polyacrylamide gel in the presence of 0.1% SDS. Protein bands were visualized by staining with Coomassie Brilliant Blue G‐250. Protein quantification of crude extracts, Affinity‐enriched fraction, and concentrated samples was performed using the bicinchoninic acid (BCA) assay (Sigma‐Aldrich), according to the manufacturer's instructions. Enzymatic activity was determined from trypsin‐like activity assays, whereas specific activity was expressed as units of enzymatic activity per milligram of protein (U mg^−1^). Purification fold was calculated as the ratio of the specific activity of each purification step to that of the crude extract, while yield (%) was determined by the recovery of total enzymatic activity after purification.

#### 2.2.3 Determination of the Inhibition Constant

To determine *K_i_* values, inhibition kinetic assays were performed using 0.1 M Tris‐HCl buffer (pH 8.2) supplemented with 20 mM CaCl_2_. The synthetic substrate Nα‐benzoyl‐l‐arginine p‐nitroanilide (l‐BApNA) (Erlanger et al. 1961; PATARROYO‐VARGAS et al. 2020) was used at concentrations of 0.1, 0.2, 0.4, 0.6, 0.8, and 1.0 mM. Assays were conducted in the presence of the inhibitor benzamidine (≥ 99% purity; Sigma‐Aldrich) at concentrations of 10, 20, 30, 40, and 50 μM, and the synthetic peptides GORE3, GORE4, and GORE5 (AminoTech, Brazil), synthesized by solid‐phase peptide synthesis (SPPS). Peptide purity (> 99%) was confirmed by reversed‐phase high‐performance liquid chromatography (RP‐HPLC), and molecular masses were verified by mass spectrometry in accordance with the manufacturer's quality control analysis. The peptides were evaluated at concentrations of 100, 200, 300, 400, and 500 μM.

### 2.3 Data Analysis

Kinetic parameters, including V_Max_, K_M_, apparent V_MAX_ (V_Maxapp_), apparent K_M_ (K_Mapp_), and inhibition constants (K_i_), were determined by nonlinear and linear regression analyses using OriginLab Origin 9.0 software (OriginLab Corporation, Northampton, MA, USA). Nonlinear regression fitting of the Michaelis–Menten equation was primarily used to estimate kinetic parameters from the experimental data. Lineweaver–Burk double‐reciprocal plots and Dixon plots were subsequently used as complementary graphical approaches to confirm the inhibition model and validate the competitive inhibition pattern observed in the nonlinear analyses. Although linearized models may introduce greater sensitivity to experimental error at low substrate concentrations, they were employed only for qualitative confirmation of the inhibition mechanism and not as the primary method for parameter estimation. Nonlinear fitting procedures were performed using least‐squares regression implemented in OriginLab Origin 9.0. All assays were conducted in three independent experiments, and the results are expressed as mean ± standard deviation. Experimental variability was minimized through independent replicates and evaluation of the consistency of fitted kinetic curves across substrate and inhibitor concentrations. Because kinetic parameters were derived from fitted enzymatic models rather than from direct independent measurements, the analyses focused primarily on characterizing and comparing inhibition patterns and fitted parameter estimates.

## 3 Results

### 3.1 In Silico Analysis

The trypsin‐like sequences of *A. gemmatalis* and *S. frugiperda*, when compared to bovine trypsin (*B. taurus*), showed sequence identities of 30.8% and 38.8%, respectively, whereas the human trypsin (*H. sapiens*) exhibited 74.9% identity relative to the bovine enzyme. Direct comparison between *S. frugiperda* and *A. gemmatalis* sequences revealed approximately 30% identity. Multiple sequence alignment (Figure 1) demonstrated that regions corresponding to the S4–S3' binding subsites are conserved among all analyzed trypsins, as well as the essential catalytic residues involved in tryptic hydrolysis.

**Figure 1:**
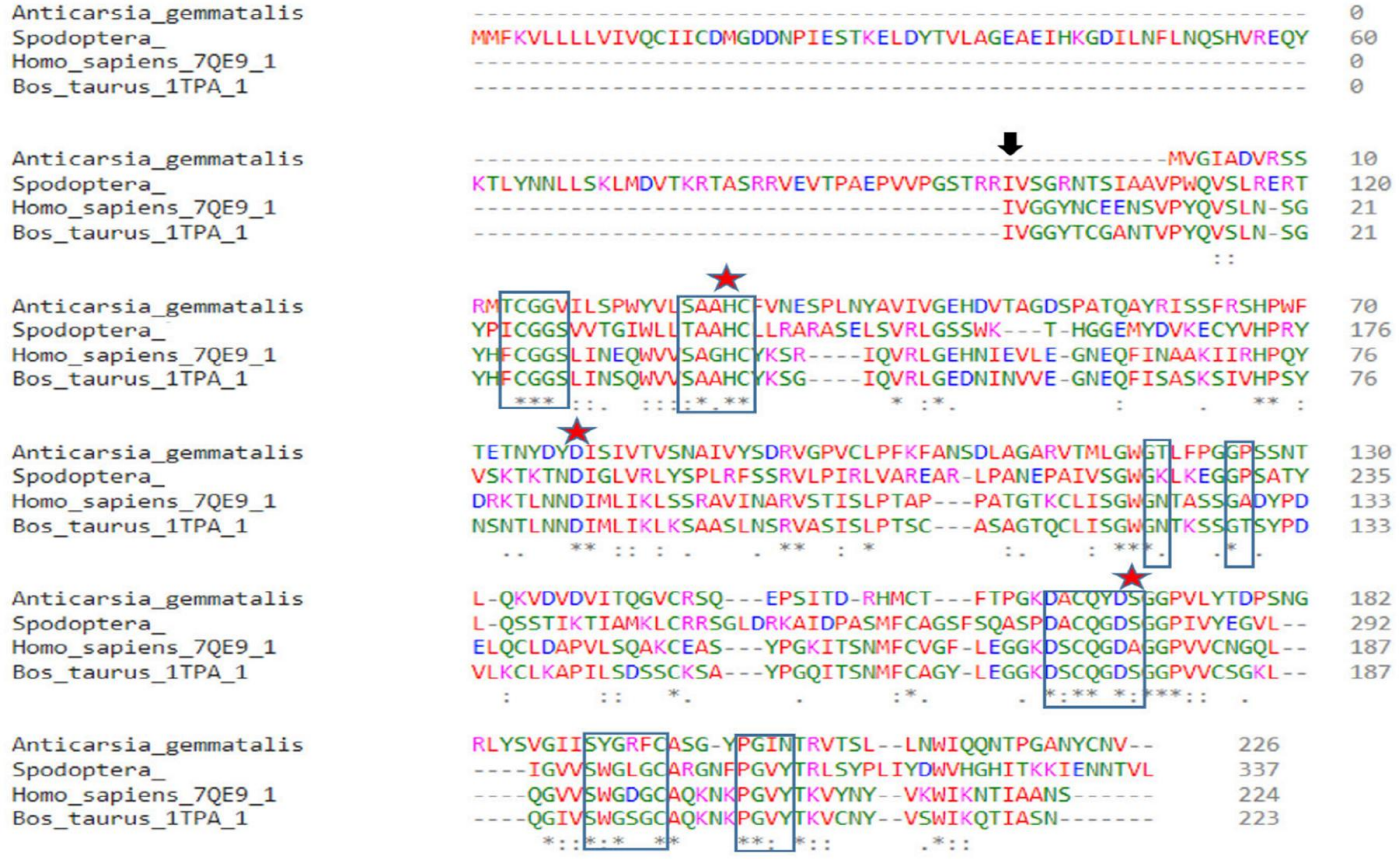
Multiple sequence alignment of trypsin sequences was performed using Clustal Omega with default parameters. The analyzed sequences correspond to *Homo sapiens* (PDB: 7QE9), *Bos taurus* (PDB: 1TPA), *A. gemmatalis*, and *S. frugiperda*. The beginning of the cleaved and catalytically active enzyme form is indicated by an arrow (↓). Enzymatic subsites S4–S3′ are highlighted by boxed regions delimiting the respective segments. Catalytic residues are indicated by asterisks positioned above the sequence alignment.

The three‐dimensional models of *A. gemmatalis* and *S. frugiperda* trypsins exhibited parameters consistent with reliable structural models (Table 1). ERRAT scores were above the minimum recommended threshold for acceptable models (> 50), while VERIFY‐3D analysis indicated appropriate compatibility between the predicted three‐dimensional structures and their respective primary sequences. Stereochemical evaluation using PROCHECK showed that more than 90% of residues were located within allowed regions of the Ramachandran plot (Table 1). Furthermore, WHATCHECK analysis did not reveal significant structural inconsistencies, suggesting proper residue packing and overall structural stability.

**Table 1:** Structural validation of three‐dimensional models generated by homology modeling with the Phyre2 server and their corresponding template structures, evaluated using ERRAT, VERIFY‐3D, PROCHECK, and WHATCHECK.

Structure	ERRAT (%)	VERIFY‐3D (%)	PROCHECK (%)	WHATCHECK
*Anticarsia gemmatalis*	58.49	94.14	96.70	12
*Spodoptera frugiperda*	53.81	88.11	96.00	13
*A. gemmatalis* (molde)	77.20	83.74	97.20	32
*S. frugiperda* (molde)	99.15	100	99.70	13

Ramachandran plot analysis generated by PROCHECK indicated that, for the modeled *A. gemmatalis* structure, 73.5% of amino acid residues were in the most favored regions, while 21.4% were positioned in additionally allowed regions (Figure 2A). For the modeled *S. frugiperda* structure, 74.6% of residues were observed in the most favored regions and 21.4% in additionally allowed regions (Figure 2B). No residues were detected in disallowed regions for *A. gemmatalis*, whereas only one residue (0.5%) was in disallowed regions in the *S. frugiperda* model. Collectively, these results indicate satisfactory stereochemical quality and structural reliability of the generated models, supporting their suitability for subsequent structural and docking analyses.

**Figure 2:**
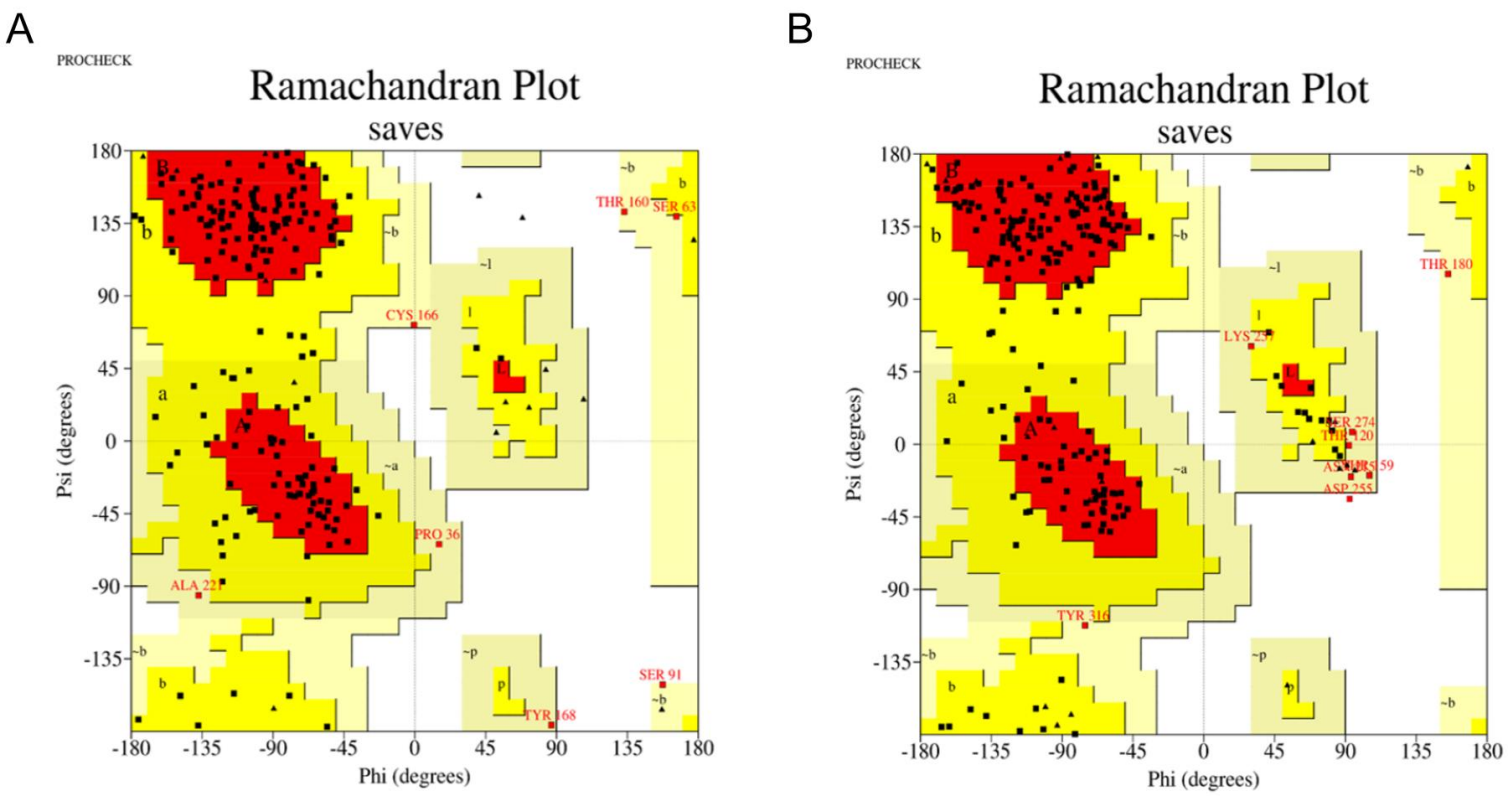
Structural validation of the modeled trypsins from *Anticarsia gemmatalis* and *Spodoptera frugiperda* using PROCHECK. (A) Ramachandran plot of the modeled *A. gemmatalis* enzyme. (B) Ramachandran plot of the modeled *S. frugiperda* enzyme.

### 3.2 Molecular Docking Analysis of Previously Described Peptides

For *B. taurus* trypsin, free binding energy values of −17.1 and −16.7 kcal mol^−1^ were observed, corresponding to estimated dissociation constants (Kd) of 0.291 pM and 0.572 pM for BPTI and SKTI, respectively. The trypsin‐like enzyme from *A. gemmatalis* exhibited free binding energies of −14.5 and −10.6 kcal mol^−1^, corresponding to estimated Kd values of 23.5 pM and 17.0 nM for BPTI and SKTI, respectively. For *S. frugiperda* trypsin, free binding energies of −11.0 and −16.0 kcal mol^−1^ were obtained, corresponding to estimated Kd values of 8.62 nM and 1.87 pM, respectively. Finally, for *H. sapiens* trypsin, free binding energy values of −14.6 and −15.4 kcal mol^−1^ were observed, corresponding to estimated Kd values of 19.8 pM and 5.14 pM for BPTI and SKTI, respectively (Table 2). The protein–peptide docking results between the modeled trypsins and the peptides GORE1 and GORE2 are presented in Table 3. Binding energies ranged from −5.20 to −7.00 kcal mol^−1^. The lowest theoretical K_i_ value was observed for the interaction between *H. sapiens* trypsin and GORE2 (7.33 × 10^−6^ M), whereas the highest theoretical K_i_ value was observed for the interaction between *A. gemmatalis* trypsin and GORE1 (1.54 × 10^−4^ M).

**Table 2:** Molecular docking results of protein–protein interactions between trypsins from *Bos taurus*, *A. gemmatalis*, *S. frugiperda*, and *H. sapiens* and the classical protein inhibitors BPTI and SKTI.

Enzyme	BPTI		SKTI	
	ΔG (kcal mol^−1^)–BPTI	Kd (M)–BPTI	ΔG (kcal mol^−1^)–SKTI	Kd (M)–SKTI
*B. taurus*	−17.1	2.91 × 10^−13^	−16.7	5.72 × 10^−13^
*A. gemmatalis*	−14.5	2.35 × 10^−11^	−10.6	1.70 × 10^−8^
*S. frugiperda*	−11.0	8.62 × 10^−9^	−16.0	1.87 × 10^−12^
*H. sapiens*	−14.6	1.98 × 10^−11^	−15.4	5.14 × 10^−12^

**Table 3:** Molecular docking analysis of protein–peptide interactions between trypsins from *Bos taurus*, *A. gemmatalis*, *S. frugiperda*, and *H. sapiens* and the peptides GORE1 and GORE2, showing binding energy (ΔG) values and theoretical inhibition constants (*K_i_*).

Enzyme	*ΔG* (kcal mol^−1^) – GORE1	*K_i_* (*M*) – GORE1	*ΔG* (kcal mol^−1^) – GORE2	*K_i_* (*M*) – GORE2
*B. taurus*	−6.20	2.91 × 10^−5^	−6.80	1.05 × 10^−5^
*A. gemmatalis*	−5.20	1.54 × 10^−4^	−5.60	7.89 × 10^−5^
*S. frugiperda*	−6.10	3.40 × 10^−5^	−6.70	1.21 × 10^−5^
*H. sapiens*	−5.70	6.64 × 10^−5^	−7.00	7.33 × 10^−6^

### 3.3 Molecular Docking Analysis of Newly Designed Peptides

Molecular docking analyses indicated that peptides Pep2 and Pep6 exhibited the lowest inhibition constant (*K_i_*) values (Table Supporting Information S1) against *A. gemmatalis* trypsin and were therefore selected for subsequent structural optimization. Based on these sequences, three additional peptides (Pep9–Pep11) were designed. Among them, Pep11 displayed *K_i_* values of 8.7 µM for *A. gemmatalis* and 5.2 µM for *S. frugiperda* and was selected for further rational truncation. This strategy led to the generation of four new peptides (Pep12–Pep15), which were subsequently evaluated by molecular docking. Among the final derivatives, Pep14 exhibited the most favorable predicted interaction profile, with binding energies of −7.4 and −7.5 kcal mol^−1^ for *A. gemmatalis* and *S. frugiperda* trypsins, respectively, and corresponding estimated K_i_ values of 3.7 µM and 3.1 µM. Structural analysis predicts interactions with residues His‐63, Gln‐197, Gly‐198, and Ser‐200, located within the enzyme's catalytic‐site region. All peptides Pep12–Pep15 exhibited binding patterns within the same region of the trypsin active site (Figure 3). Peptide Pep12, which retained tyrosine as the C‐terminal residue, was designated GORE3. Based on this sequence, site‐directed substitutions were introduced at the P1 position, resulting in the generation of two additional peptides, GORE4 and GORE5.

**Figure 3:**
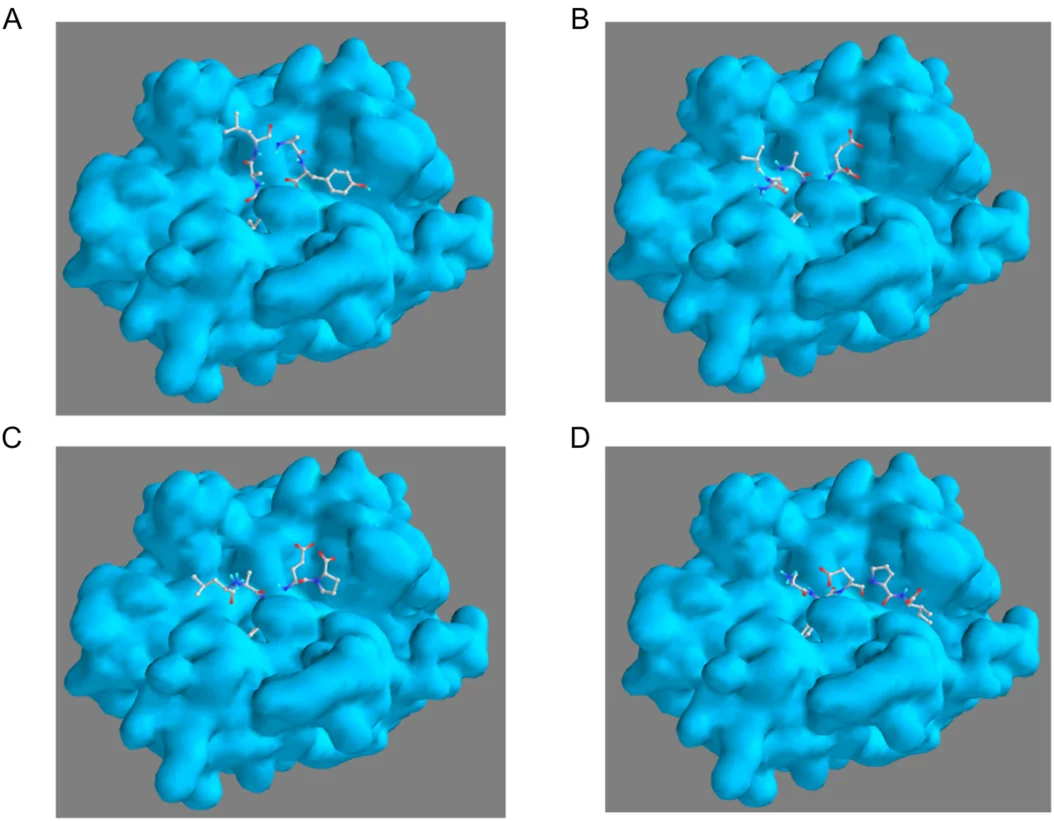
Molecular docking representation of *Bos taurus* trypsin in complex with the inhibitory peptides Pep12 (A), Pep13 (B), Pep14 (C), and Pep15 (D).

Protein–peptide docking of GORE3 (A), GORE4 (B), and GORE5 (C) against the modeled trypsin‐like enzyme from *A. gemmatalis*, performed using the ClusPro 2.0 server, generated multiple interaction clusters (Figure 4). The selected representative complexes show distinct interaction patterns in the 2D interaction maps. GORE3 (A) exhibited a mixed interaction profile, including several conventional hydrogen bonds (e.g., with CYS A:93, GLY A:245, SER A:247, ALA A:46, and ALA A:43), a π‐sulfur interaction with CYS A:93, and multiple hydrophobic contacts (alkyl/π‐alkyl) involving residues such as PRO A:45, HIS A:92, PRO A:222, ILE A:221, and LEU A:24. GORE4 (B) formed an extended hydrogen‐bonding network with residues including GLY A:245, SER A:76, CYS A:77, ARG A:139, GLN A:244, and ALA A:43, together with an attractive charge interaction with GLU A:219 and alkyl contacts with PRO A:222, PRO A:41, and LEU A:24. In contrast, GORE5 (C) showed a more electrostatically driven interaction pattern, characterized by a salt bridge and an attractive charge interaction with GLU A:219, conventional hydrogen bonds with PRO A:41, ALA A:43, and ARG A:139, a carbon hydrogen bond with PRO A:222, and an alkyl contact with ALA A:46. The reported values (−805.0, −639.5, and −723.6) correspond to the weighted energy scores used by ClusPro for cluster ranking and should not be interpreted as absolute binding free energies (ΔG, kcal/mol). Instead, these scores were used to compare the peptides evaluated and identify the most energetically favorable complexes (Table 4).

**Figure 4:**
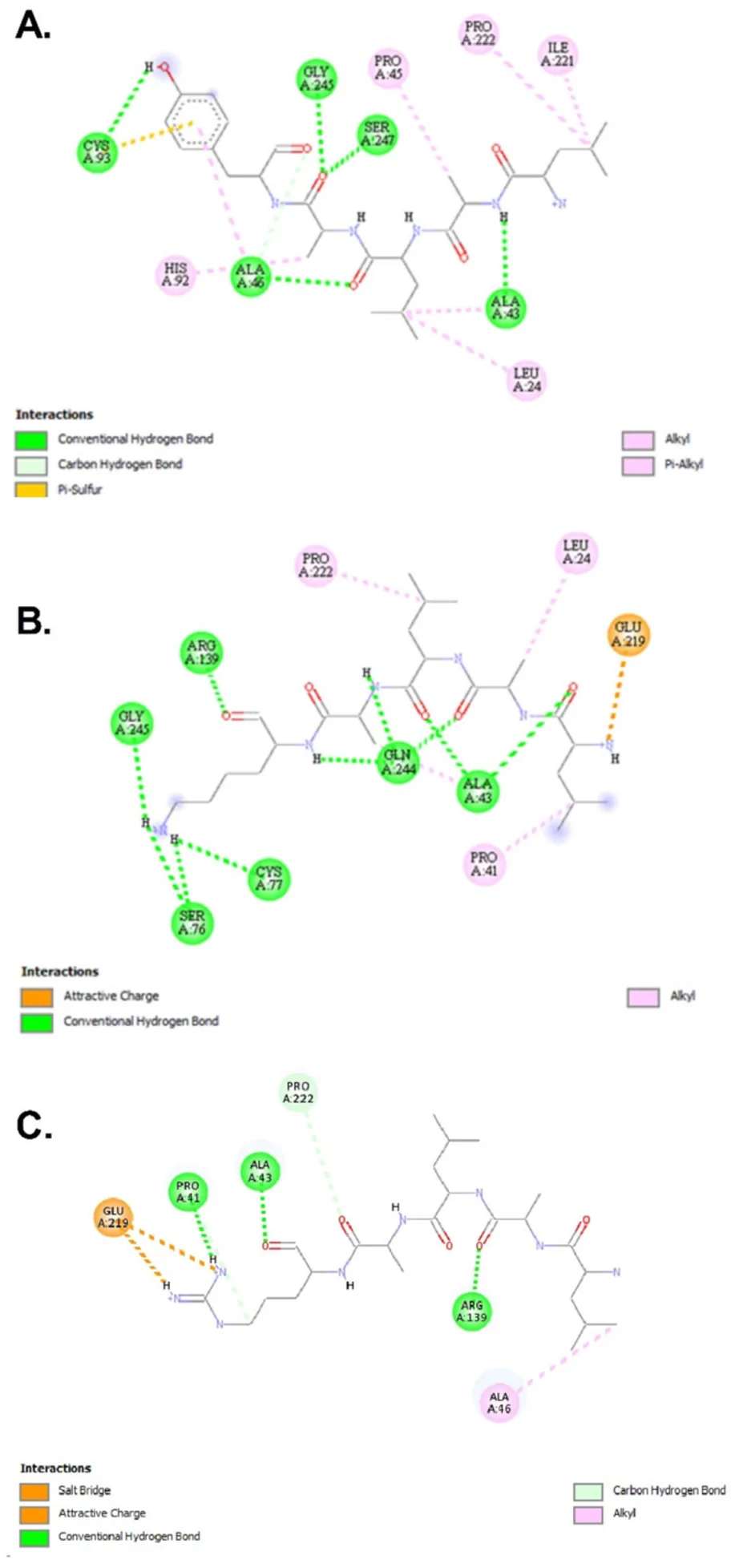
Molecular docking complexes between *A. gemmatalis* trypsin‐like enzymes and inhibitory peptides. (A) Predicted enzyme–peptide complex with GORE3; (B) predicted enzyme–peptide complex with GORE4, and (C) Predicted enzyme–peptide complex with GORE5, illustrating the binding mode within the catalytic region.

**Table 4:** Molecular docking results obtained with ClusPro 2.0 for interactions between *A. gemmatalis* trypsin‐like enzyme and GORE3, GORE4, and GORE5, showing the number of cluster members, cluster center score, and lowest weighted score for each complex.

Peptide	Cluster members	Cluster center score	Lowest weighted score
GORE3	521	−660.1	−805.0
GORE4	939	−541.1	−639.5
GORE5	442	−659.0	−723.6

*Note:* ClusPro scores are weighted docking scores expressed in arbitrary scoring units and are intended only for relative ranking of docking clusters. They do not represent thermodynamic binding free energies (ΔG, kcal/mol) and should not be directly compared with AutoDock Vina ΔG values.

## 4 Results of In Vitro Assays

### 4.1 Enrichment of Midgut Trypsin‐Like Activity From *A. gemmatalis*

Affinity chromatography on Benzamidine‐Sepharose generated a fraction enriched in trypsin‐like activity, as indicated by the increase in specific activity toward the trypsin substrate l‐BApNA. Specific activity increased from 3.83 × 10^−5^ U mg^−1^ in the crude extract to 2.40 × 10^−4^ U mg^−1^ in the affinity‐enriched fraction, corresponding to a 6.28‐fold enrichment. Total enzymatic activity decreased from 0.3006 to 0.0766 U, resulting in a recovery of 25.47% (Table 5). These results demonstrate enrichment of enzymatic activity with biochemical characteristics consistent with trypsin‐like proteases but do not, by themselves, establish the molecular identity of the proteins present in the fraction.

**Table 5:** Enrichment of trypsin‐like activity from *A. gemmatalis* midgut extract by affinity chromatography on Benzamidine‐Sepharose.

Purification step	Total protein (mg)	Total activity (U)	Specific activity (U mg^−1^)	Purification fold	Yield (%)
Crude extract	7,852.61	0.3006	3.83 × 10^−5^	1.00	100.00
Purified fraction	318.70	0.07656	2.40 × 10^−4^	6.28	25.47

*Note:* Specific activity (U·mg^−1^) was defined as the amount of enzyme releasing 1 µmol of product per minute under the experimental conditions. The purification fold was calculated as the ratio of the specific activity of the Affinity‐enriched fraction to that of the crude extract, and yield (%) was determined from the total recovered activity relative to the crude extract.

After affinity chromatography, SDS‐PAGE showed a reduction in protein‐band complexity relative to the crude midgut extract (Figure 5). Previous studies of *A. gemmatalis* have reported soluble and membrane‐associated trypsin‐like proteases with apparent molecular masses within approximately 25–35 kDa following affinity purification with p‐aminobenzamidine‐based matrices (Patarroyo‐Vargas et al. 2018; [additional reference]). However, because protein identity was not confirmed by mass spectrometry in the present study, individual SDS‐PAGE bands cannot be unequivocally assigned to trypsin isoforms. Therefore, the chromatographic fraction is conservatively referred to here as a trypsin‐like activity‐enriched fraction, based on its affinity for Benzamidine‐Sepharose and its hydrolytic activity toward l‐BapNA.

**Figure 5:**
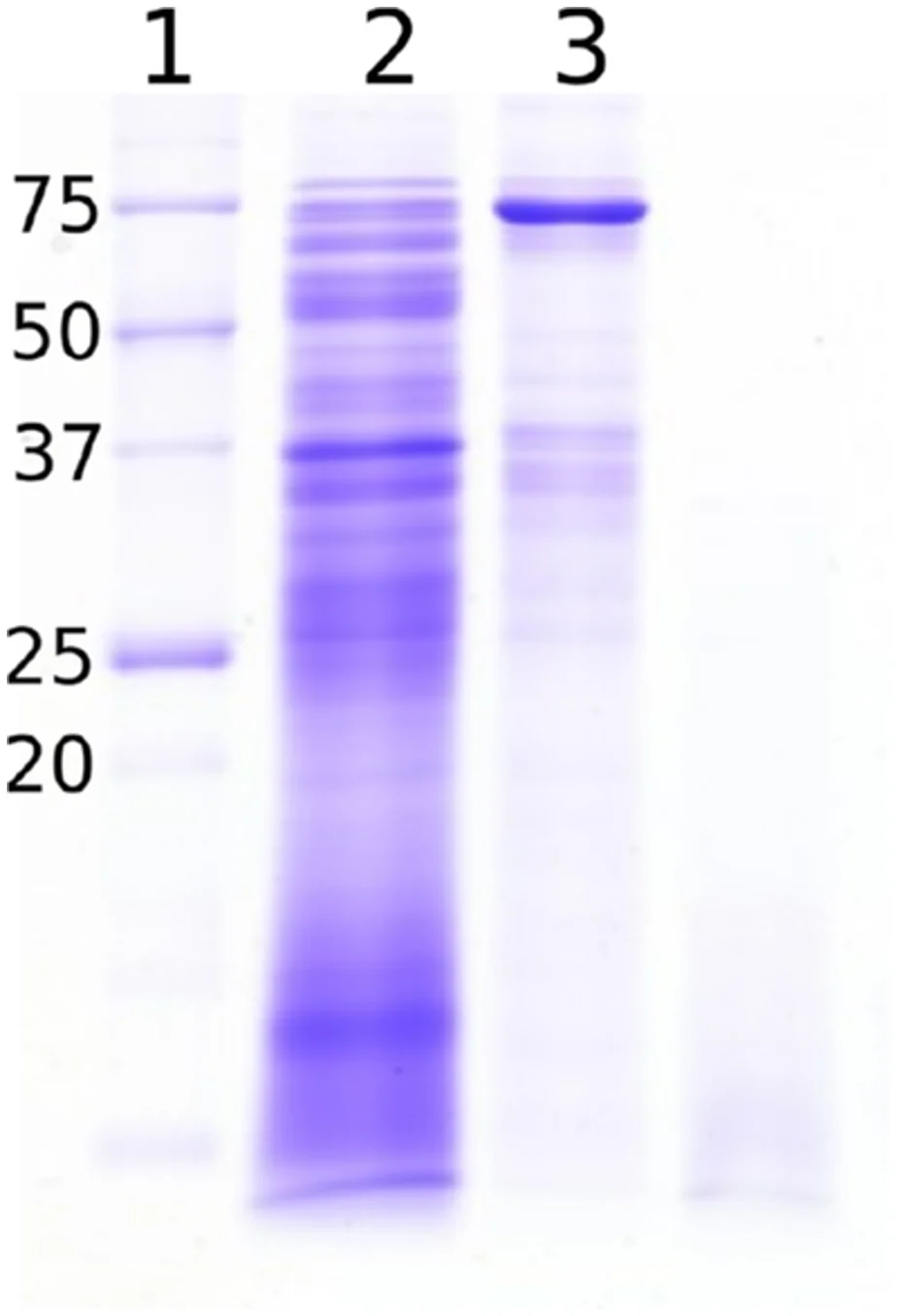
SDS‐PAGE electrophoretic profile of partially purified midgut trypsin‐like enzymes from *Anticarsia gemmatalis*. (1) Molecular weight marker; (2) crude midgut extract; (3) fraction obtained after affinity chromatography on Benzamidine‐Sepharose.

#### 4.1.1 Enzymatic Characterization: Inhibition Kinetics

The kinetic parameters obtained for bovine trypsin and for *A. gemmatalis* trypsin‐like enzymes in the presence of inhibitors are presented in Table 6. For bovine trypsin, *V*_max_ values ranged from 107.97 to 250.96 nM·s^−1^, whereas *K_M_* values ranged from 0.104 to 0.670 mM. For *A. gemmatalis* trypsin‐like enzymes, apparent *V*_max_ (*V*_maxapp_) values ranged from 42.32 to 218.37 nM·s^−1^, and apparent *K_M_* (*K*_Mapp_) values ranged from 0.070 to 0.598 mM. Tryptic activity exhibited a hyperbolic velocity curve as a function of substrate concentration, consistent with the Michaelis–Menten kinetic model, within the range of 0.1 to 1 mM l‐BApNA for bovine trypsin (Figure 6). A similar kinetic behavior was observed for *A. gemmatalis* trypsin‐like enzymes (Figure 7). Linearization of the curves according to the Lineweaver–Burk double‐reciprocal model, as shown in the figure insets, confirmed the suitability of the Michaelis–Menten model for describing the experimental data. For inhibition kinetics, different substrate and inhibitor concentrations were evaluated. The Lineweaver–Burk plots revealed a pattern consistent with competitive inhibition, characterized by a progressive increase in slope as inhibitor concentration increases, while the y‐intercept remains constant. Secondary slope plots were constructed using the angular coefficients obtained from the Lineweaver–Burk plots for bovine trypsin (Figure Supporting Information S1) and *A. gemmatalis* trypsin‐like enzymes (Figure Supporting Information S2) to further validate the inhibition model. The linear relationship observed between slopes and inhibitor concentrations confirmed competitive inhibition. Dixon plots, constructed by varying inhibitor concentrations at different fixed substrate concentrations, also exhibited a pattern consistent with competitive inhibition. The lines converged at a common point on the x‐axis for both bovine trypsin (Figure 8) and A. gem*matalis* trypsin‐like enzymes (Figure 9), further supporting the competitive inhibition mechanism. Benzamidine exhibited K_i_ values of 0.026 mM for bovine trypsin and 0.0139 mM for *A. gemmatalis* trypsin‐like enzymes. GORE3 showed Ki values of 2.08 and 1.80 mM for bovine trypsin and *A. gemmatalis* trypsin‐like enzymes, respectively. GORE4 presented Ki values of 1.90 and 1.63 mM, respectively. Among the evaluated peptides, GORE5 exhibited the lowest K_i_ values, with 1.07 mM against bovine trypsin and 0.78 mM against *A. gemmatalis* trypsin‐like enzymes (Table 6).

**Table 6:** Inhibition constants (K_i_) of bovine trypsin and partially purified midgut trypsin‐like enzymes from *A. gemmatalis* in the presence of benzamidine and the peptides GORE3, GORE4, and GORE5. K_i_ values (mM) were calculated from kinetic analyses based on a competitive inhibition model.

Inhibitor	*Kᵢ* (mM)–bovine trypsin	*Kᵢ* (mM)–*A. gemmatalis*
GORE3	2.08 ± 0.12	1.80 ± 0.09
GORE4	1.90 ± 0.10	1.63 ± 0.08
GORE5	1.07 ± 0.05	0.78 ± 0.04

**Figure 6:**
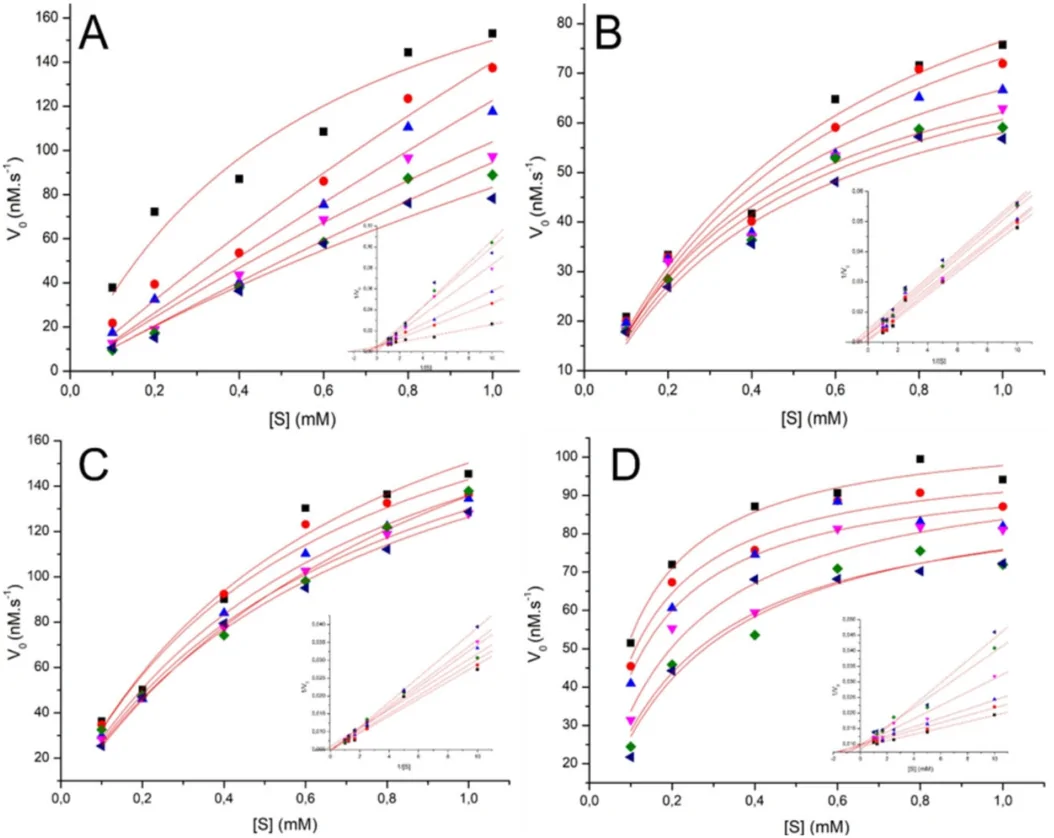
Michaelis–Menten curves for inhibition kinetics of bovine trypsin in the presence of inhibitors: (A) benzamidine, (B) GORE3, (C) GORE4, and (D) GORE5.

**Figure 7:**
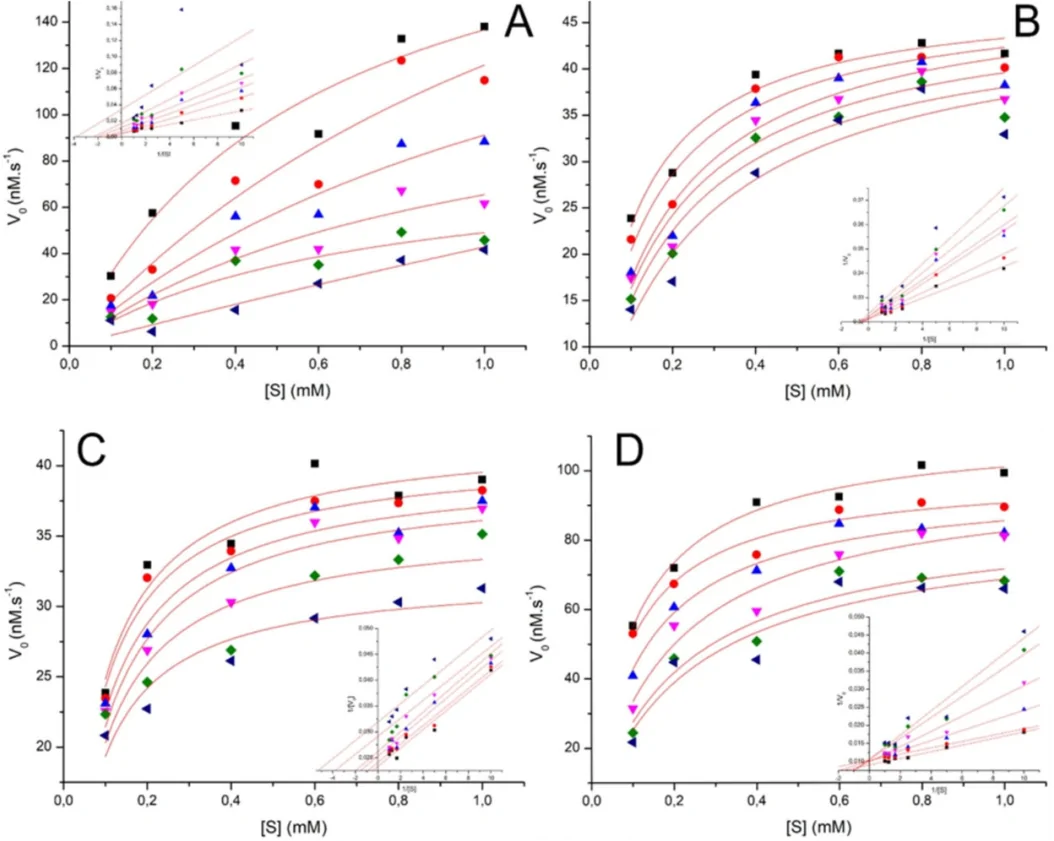
Michaelis–Menten curves for inhibition kinetics of partially purified *Anticarsia gemmatalis* trypsin‐like enzymes in the presence of inhibitors: (A) benzamidine, (B) GORE3, (C) GORE4, and (D) GORE5.

**Figure 8:**
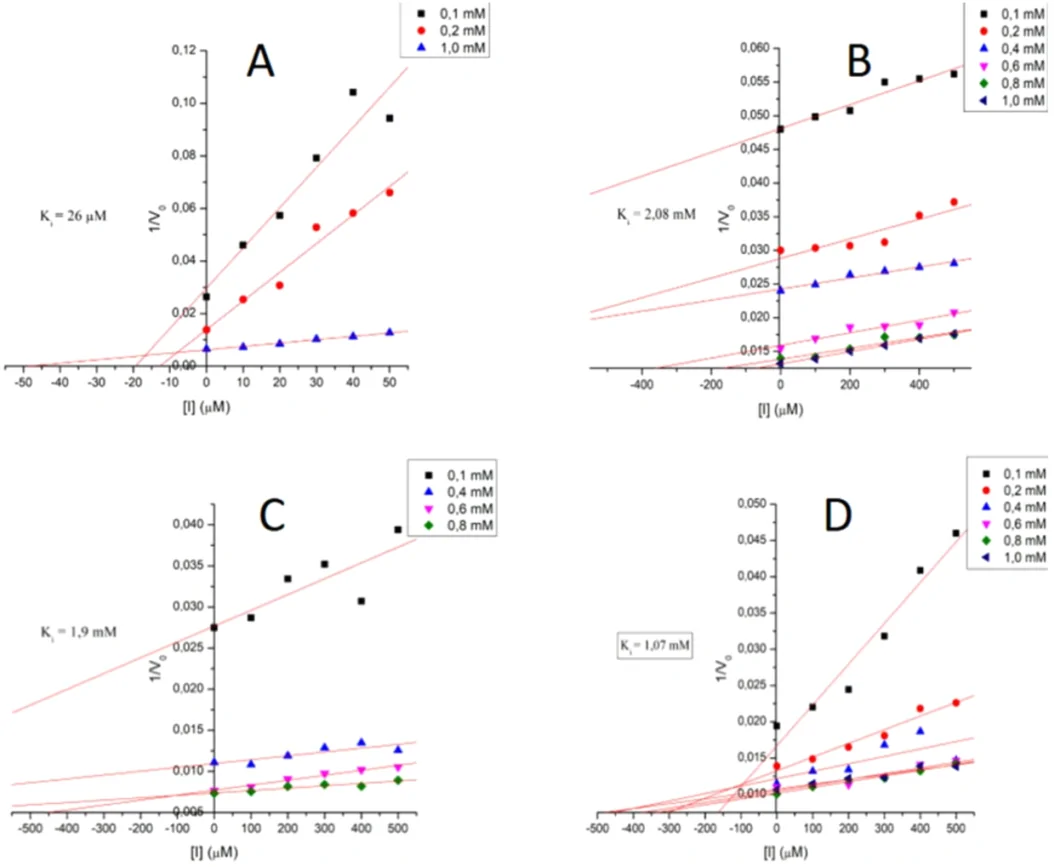
Dixon plots for inhibition of bovine trypsin in the presence of inhibitors: (A) benzamidine, (B) GORE3, (C) GORE4, and (D) GORE5.

**Figure 9:**
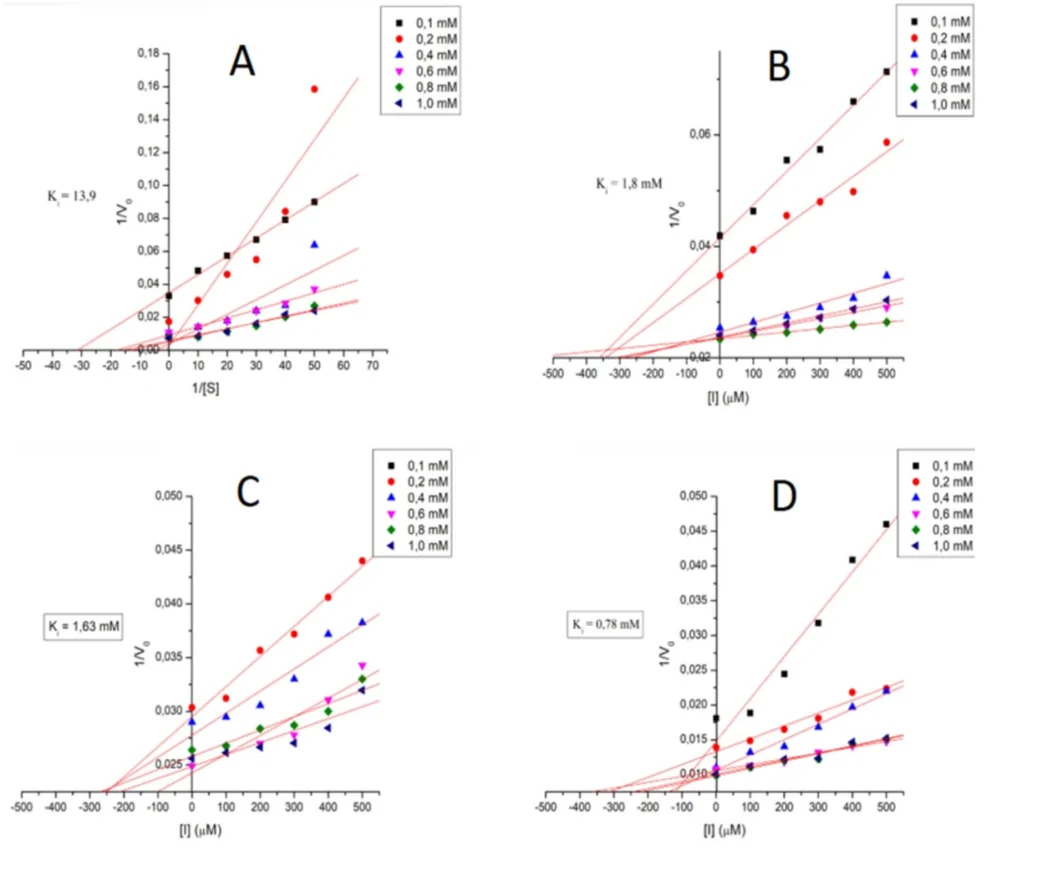
Dixon plots for inhibition of partially purified *Anticarsia gemmatalis* trypsin‐like enzymes in the presence of inhibitors: (A) benzamidine, (B) GORE3, (C) GORE4, and (D) GORE5.

## 5 Discussion

In the present study, sequence alignment demonstrated conservation of key residues involved in substrate binding (Asp‐194, Ser‐195, Gln‐197, and Gly‐198) and of the classical catalytic triad (His‐63, Asp‐107, and Ser‐200) across all analyzed trypsins (Ribeiro et al. 2020). The S4–S3′ subsites were likewise conserved (Hanisch et al. 1987; Marana et al. 2002), supporting preservation of the canonical catalytic mechanism of trypsin‐like serine proteases. Despite this functional conservation, the relatively low global sequence identity (30.8%–38.8%) between insect and mammalian enzymes suggests structural divergence that may influence active‐site topology and inhibitor specificity (Hemmati, Karam Kiani et al. 2021).

The three‐dimensional models predicted by Phyre2 exhibited validation parameters consistent with reliable structures for docking analyses. Ramachandran plots showed that more than 96% of residues were in the allowed regions, and additional validation tools (ERRAT, VERIFY‐3D, and WHATCHECK) supported the structural robustness of the models. These results justify their application in subsequent bioinformatic analyses (Laskowski et al. 1993).

Docking‐derived Kd values differed from experimentally reported affinity values for BPTI and SKTI. For bovine trypsin, the calculated Kd values (0.29 and 0.57 pM for BPTI and SKTI, respectively) differed substantially from the corresponding experimentally reported values (Yang et al. 2021; Xu et al. 2020). Similarly, for *A. gemmatalis*, discrepancies were observed when compared with previously reported values (de Almeida Barros et al. 2021). These differences reflect intrinsic limitations of docking‐based affinity estimation, which does not fully account for solvent effects, protein flexibility, and entropic contributions. Therefore, docking‐derived affinity estimates should be interpreted primarily for comparative purposes rather than as accurate quantitative predictions of experimental binding affinity.

Notably, predicted *K_i_* values for the synthetic peptides GORE1 and GORE2 showed greater agreement with previously reported experimental data, suggesting improved predictive consistency for smaller, more flexible ligands. This observation supports the rationale for exploring short peptide scaffolds derived from regulatory regions.

The rational truncation strategy progressively improved the docking‐derived affinity estimates, and the pentapeptides Pep12–Pep15 emerged as the most favorable candidates according to the in silico screening, with theoretical Ki values in the low‐micromolar range. However, these docking‐derived Ki values substantially underestimated the experimentally determined inhibition constants. Whereas the in silico screening predicted affinities in the low‐micromolar range, kinetic assays of the subsequently evaluated GORE peptides yielded Ki values of 1.80, 1.63, and 0.78 mM for GORE3, GORE4, and GORE5, respectively. Thus, the experimental affinities were approximately two orders of magnitude weaker than suggested by the docking‐derived estimates. This discrepancy demonstrates that the calculated Ki values should be interpreted as comparative theoretical estimates for candidate prioritization rather than as quantitative predictions of experimental affinity.

Protein–peptide interaction analysis revealed preferential binding within the catalytic region, involving residues His‐63, Gln‐197, Gly‐198, and Ser‐200. Direct interaction with catalytic‐site residues supports a mechanism of competitive inhibition. Additionally, the absence of lysine or arginine residues, preferred cleavage sites for trypsins, combined with the presence of proline, which may confer resistance to proteolytic cleavage, reinforces the interpretation that these peptides act as inhibitors rather than substrates (Simpson 2006).

The competitive inhibition pattern suggests that the peptides occupy regions close to the catalytic pocket, thereby impairing substrate access to the active site (Perona and Craik 1995; Oliveira et al. 1993). Interactions involving residues associated with the S1 specificity region likely contribute to stabilization of the peptide–enzyme complex through electrostatic and hydrogen‐bond interactions (Perona and Craik 1995). In addition, peptide accommodation within the catalytic region may generate steric hindrance that interferes with proper substrate positioning during catalysis (Otlewski et al. 2005). These combined structural effects are consistent with the experimentally observed reduction in trypsin‐like activity and reinforce the proposed mechanism of competitive inhibition.

Among the evaluated candidates, Pep12 was selected and designated GORE3 due to its reduced size and favorable physicochemical characteristics. Site‐directed substitutions at the P1 position generated GORE4 and GORE5. Although GORE3 exhibited the most favorable docking score in the in silico analyses, kinetic assays showed that GORE5 had the lowest Ki among the evaluated peptides. However, its Ki remained within the millimolar range, indicating weak inhibitory affinity despite the relative improvement over GORE3 and GORE4. This quantitative discrepancy highlights a major limitation of docking‐based affinity estimation. Docking scoring functions provide simplified approximations of molecular recognition and do not fully account for solvent effects, protein and peptide conformational flexibility, entropic contributions, protonation states, or structural rearrangements occurring during binding and catalysis (Paulo, Schneider, et al. 2026; $author1$ et al.). Consequently, the docking‐derived Ki values did not accurately reproduce the absolute experimental affinity of this peptide series.

Nevertheless, the computational analysis retained qualitative mechanistic value. The predicted peptide poses were preferentially located within or near the catalytic region, and the subsequent kinetic experiments independently demonstrated a competitive inhibition mechanism. Therefore, the main predictive strength of the in silico workflow in the present study was the identification of plausible binding regions and the prioritization of peptide candidates, rather than the accurate quantitative prediction of absolute Ki values. Experimental kinetic measurements remain essential for establishing inhibitory potency.

Kinetic characterization confirmed that purified enzymes exhibit typical trypsin‐like behavior, with *K_M_* values consistent with literature reports (da Silva Júnior et al. 2020; Nakasu et al. 2014). All graphical analyses (Michaelis–Menten, Lineweaver–Burk, and Dixon plots) consistently supported a competitive inhibition mechanism for GORE3, GORE4, and GORE5. Benzamidine *K_i_* values obtained in this study were comparable to previously reported values, confirming assay reliability.

Despite the competitive inhibition demonstrated experimentally, the Ki values obtained for GORE3, GORE4, and GORE5 against *A. gemmatalis* trypsin‐like enzymes (1.80, 1.63, and 0.78 mM, respectively) indicate relatively weak inhibitory affinity. This represents an important limitation for direct agricultural application of the current peptide sequences. In vivo, maintaining peptide concentrations in the millimolar range within the insect midgut would be difficult because the effective inhibitor concentration would be affected by dilution after ingestion, proteolytic degradation, intestinal transit, and limitations associated with peptide formulation and delivery. Moreover, because these peptides act through a competitive mechanism, their inhibitory effectiveness in vivo would also depend on the local concentration of dietary protein substrates and digestive proteases. Therefore, the present peptides should be considered proof‐of‐concept molecular scaffolds rather than immediately deployable bioinsecticidal compounds.

Future optimization should consequently focus on simultaneously increasing target affinity and proteolytic stability. Strategies may include the incorporation of non‐natural or d‐amino acids at positions that do not disrupt residues essential for enzyme recognition, N‐ or C‐terminal modifications, and rational replacement of residues interacting with the S1 and neighboring subsites to strengthen electrostatic, hydrogen‐bonding, and hydrophobic interactions. Peptide cyclization, including head‐to‐tail or side‐chain cyclization, could further restrict conformational flexibility and increase resistance to exopeptidases and endopeptidases. Other conformational‐constraining approaches, including peptide stapling where structurally compatible, may also be explored to improve metabolic stability and binding. These optimized derivatives should subsequently be evaluated through kinetic assays, proteolytic‐stability studies under simulated or isolated insect midgut conditions, and ultimately in vivo feeding assays before their practical potential for pest control can be established.

## 6 Conclusions

The application of bioinformatics approaches proved to be an efficient and cost‐effective strategy for the rational prospection of peptides with inhibitory potential, enabling the screening of multiple sequences and the selection of promising candidates for experimental validation. Through this strategy, 15 peptides were designed with predicted interaction profiles and theoretical inhibitory potential comparable to or greater than those reported for previously described inhibitors, with peptides Pep12–15 emerging as the most promising candidates for further in silico, in vitro, and in vivo investigations. Kinetic characterization using bovine trypsin and benzamidine as reference standards confirmed the enzymatic profile of *A. gemmatalis* trypsin‐like enzymes and was consistent with competitive inhibition. The lower inhibition constant observed for benzamidine against trypsin‐like enzymes, compared with bovine trypsin, reinforces the functional similarity of these proteases to classical trypsins.

Consistently, GORE3, GORE4, and particularly GORE5 exhibited competitive inhibition, reproducing the kinetic pattern observed for benzamidine and supporting their interaction with the catalytic region of the target enzymes. Collectively, the results indicate that these peptides, especially GORE5, represent potential scaffolds for developing digestive trypsin inhibitors targeting *A. gemmatalis*. However, additional in vivo studies are still required to evaluate their effects on biological parameters, insect development, and overall life‐cycle performance, and to assess their practical feasibility under agricultural conditions.

Despite the promising inhibitory profiles observed, the peptides evaluated in this study still exhibit weak inhibitory affinity compared with classical protein inhibitors. In addition, important aspects related to peptide stability under insect gut conditions, formulation strategies, delivery efficiency, production scalability, and field applicability remain to be investigated. Future studies integrating molecular optimization, stability analyses, formulation approaches, and in vivo field evaluations will be essential to better assess the practical potential of these peptide scaffolds for agricultural applications.

## Author Contributions

**Geisiane Aparecida Mariano:** project administration; methodology; conceptualization; investigation; writing–original draft. **João Vitor Aguilar Oliveira:** conceptualization; methodology; software. **Rafael Júnior de Andrade:** methodology; validation; visualization. **Daniel Guimarães Silva Paulo:** writing–review and editing; visualization; validation; conceptualization. **Yaremis Beatriz Meriño Cabrera:** methodology; validation; formal analysis. **Eulálio Gutemberg Dias dos Santos:** formal analysis; software; validation. **Ian Lucas Batista Santos:** visualization; validation. **Milena Godoi Lima:** validation; visualization. **Maria Julia Summy:** validation; visualization. **Otávio José Bernardes Brustolini:** validation; visualization. **Neilier Rodrigues da Silva Júnior:** supervision; validation; visualization. **Humberto Josué Oliveira Ramos:** supervision; funding acquisition; validation; visualization. **Maria Goreti Almeida Oliveira:** supervision; funding acquisition; validation.

## Protein Accession Numbers

*Bos taurus* trypsin: PDB ID 1TPA.

*Homo sapiens trypsin:* PDB ID 7QE9.

*Anticarsia gemmatalis* trypsin‐like protease: transcript ID DN773_i3.

*Spodoptera frugiperda* trypsin‐like proteases: NCBI accessions XP_050552352.1, ACR25157.1, QLC28936.1, and XP_050550273.1.

Soybean Kunitz Trypsin Inhibitor (SKTI): UniProt ID P01070.

Bovine Pancreatic Trypsin Inhibitor (BPTI): UniProt ID P00974.

## Conflicts of Interest

The authors declare no conflicts of interest.

## Supporting information


Supporting File

## Data Availability

The data that support the findings of this study are available from the corresponding author upon reasonable request.
