# Supplementary material for: Trypsinogen Pro‐Region as a Molecular Scaffold for the Design of Competitive Peptide Inhibitors of Trypsin‐Like Proteases
Source: Arch Insect Biochem Physiol. 2026 Oct 4;123(2):e70224. doi: 10.1002/arch.70224 (PMC13634603; doi:10.1002/arch.70224)
Supplement: Supplementary file 1 [file ARCH-123-e70224-s001.docx]

**Table S1.** Molecular docking analysis between the selected enzymes and peptides designed from the pro-region of *A. gemmatalis*. Peptides 1–8 contain 10 amino acid residues, peptides 9–11 contain 8 residues, and peptides 12–15 contain 5 residues. The best-performing peptides from each size group were used as templates for the development of shorter derivatives. Docking analyses were performed using PyRx and AutoDock Vina with default parameters, considering the entire molecular surface for interaction screening.

| **Peptide** | **Bos taurus** |  | ***A. gemmatalis*** |  | ***S. frugiperda*** |  | ***H. sapiens*** |  |
| --- | --- | --- | --- | --- | --- | --- | --- | --- |
|  | Energy (kcal/mol) | K_i_ (mol) | Energy (kcal/mol) | K_i_ (mol) | Energy (kcal/mol) | K_i_ (mol) | Energy (kcal/mol) | K_i_ (mol) |
| Pep1 | -6.00 | 3.99501E-05 | -4.90 | 2.55803E-04 | -6.90 | 8.74470E-06 | -6.40 | 2.03369E-05 |
| Pep2 | -7.30 | 4.45157E-06 | -6.90 | 8.74470E-06 | -7.60 | 2.68280E-06 | -8.10 | 1.15358E-06 |
| Pep3 | -7.30 | 4.45157E-06 | -6.00 | 3.99501E-05 | -7.30 | 4.45157E-06 | -7.20 | 5.27012E-06 |
| Pep4 | -6.90 | 8.74470E-06 | -6.50 | 1.71782E-05 | -6.80 | 1.03527E-05 | -7.50 | 3.17612E-06 |
| Pep5 | -6.20 | 2.85037E-05 | -6.30 | 2.40765E-05 | -7.10 | 6.23920E-06 | -7.00 | 7.38647E-06 |
| Pep6 | -6.20 | 2.85037E-05 | -6.90 | 8.74470E-06 | -8.40 | 6.95223E-07 | -6.50 | 1.71782E-05 |
| Pep7 | -7.90 | 1.61683E-06 | -6.60 | 1.45101E-05 | -7.70 | 2.26611E-06 | -7.60 | 2.68280E-06 |
| Pep8 | -7.40 | 3.76015E-06 | -6.30 | 2.40765E-05 | -6.90 | 8.74470E-06 | -7.80 | 1.91414E-06 |
| Pep9 | -7.40 | 3.76015E-06 | -5.60 | 7.84783E-05 | -7.50 | 3.17612E-06 | -7.10 | 6.23920E-06 |
| Pep10 | -7.70 | 2.26611E-06 | -6.60 | 1.45101E-05 | -7.70 | 2.26611E-06 | -7.50 | 3.17612E-06 |
| Pep11 | -7.30 | 4.45157E-06 | -6.90 | 8.74470E-06 | -7.20 | 5.27012E-06 | -7.20 | 5.27012E-06 |
| Pep12 | -6.90 | 8.74470E-06 | -6.80 | 1.03527E-05 | -6.00 | 3.99501E-05 | -7.50 | 3.17612E-06 |
| Pep13 | -7.20 | 5.27012E-06 | -6.60 | 1.45101E-05 | -7.40 | 3.76015E-06 | -7.70 | 2.26611E-06 |
| Pep14 | -7.40 | 3.76050E-06 | -7.40 | 3.76050E-06 | -7.50 | 3.17612E-06 | -8.20 | 9.74407E-07 |
| Pep15 | -7.70 | 2.26611E-06 | -7.00 | 7.38647E-06 | -7.80 | 1.91414E-06 | -7.50 | 3.17612E-06 |

**Figure S1.** Slopes of the Lineweaver–Burk plots as a function of inhibitor concentration for bovine trypsin. (A) Benzamidine; (B) GORE 3; (C) GORE 4; (D) GORE 5.

**Figure S2.** Linear regression of the slopes obtained from Lineweaver–Burk plots plotted against inhibitor concentration for *A. gemmatalis* trypsin-like enzymes. (A) Benzamidine; (B) GORE 3; (C) GORE 4; (D) GORE 5.
